# Isolating Viable Ancient Bacteria: What You Put In Is What You Get Out

**DOI:** 10.1128/genomeA.00712-16

**Published:** 2016-08-25

**Authors:** Raphael Eisenhofer, Alan Cooper, Laura S. Weyrich

**Affiliations:** Australian Centre for Ancient DNA, University of Adelaide, Adelaide, Australia

## LETTER

In the recent publication “Draft Genome Sequence of *Enterococcus faecium* Strain 58m, Isolated from Intestinal Tract Content of a Woolly Mammoth, *Mammuthus primigenius*” in *Genome Announcements* (1), Goncharov et al. claim to have isolated and grown in pure culture a 28,000-year-old *Enterococcus faecium* strain. However, the authors ignored a breadth of literature about the authentication of ancient DNA, failed to adhere to recommended guidelines (2), and did not provide the appropriate experimental controls and analyses required to substantiate such a claim. Here, we present a subsequent reanalysis of the Goncharov et al. isolate and demonstrate by multilocus sequence typing (MLST) that this strain likely represents a modern contaminant.

Previous efforts aimed at isolating viable ancient bacteria have been consistently controversial (3). Viable bacteria have been reported from a 250 million-year-old salt crystal (4) and 25- to 40 million-year-old amber (5). These unlikely findings have not been independently replicated, and failed molecular phylogenetic tests (6–8). In light of such dubious claims, a set of rigorous authentication criteria have been proposed (2). These include evolutionary rates tests, whereby phylogenetic comparisons of the ancient organism with its modern counterparts are expected to show substantial genetic differences, accumulated through time.

In the Goncharov et al. study, the authors admit that *E. faecium* is a common member of the human gut community and can be found from numerous environmental sources, yet strangely they did nothing to prevent or control for modern contamination at various stages of their experiment. Modern contaminants can enter during the sampling procedure (2) or during laboratory analysis (i.e., culturing or DNA sequencing). Contamination during laboratory analysis is especially probable when the isolate is cultured using broad-spectrum media (2), as used by the authors. Clearly, the authors should have considered these factors and demonstrated or minimally investigated to determine that their isolate did not represent a modern human or environmental contaminant, something they failed to do.

To test the authenticity of the authors’ claims, we queried the genome assembly of the “ancient” *E. faecium* isolate against published sequences in the *E. faecium* MLST database (http://pubmlst.org/efaecium/), which contains >2,800 modern *E. faecium* isolates. The MLST sequence from the putatively ancient *E. faecium* isolate matches the previously identified sequence type 32 (ST32) with 100% sequence homology; this is unexpected if the genome is ancient. Modern isolates of ST32 are known from the Russian Federation, where this study took place. If the bacterium was an ancient resident of the mammoth gut, it should not be identical to a modern human isolate, given that many gut microorganisms coevolved with their hosts and that humans and mammoths diverged over 100 million years ago (9). The lack of even a single nucleotide difference within seven genetic loci, coupled with the fact that this bacterium is commonly found in the modern human gut community and other environmental sources, is damning evidence that the authors’ isolate represents a modern contaminant.

The authors’ “ancient” *E. faecium* isolate is highly similar to modern human isolates and is therefore almost certainly not an ancient mammoth strain.
